# Improvement of SAW Resonator Performance by Petal-like Topological Insulator

**DOI:** 10.3390/s24175584

**Published:** 2024-08-28

**Authors:** Jin Bai, Lixia Li, Chenyang Chai

**Affiliations:** School of Mechanical and Electrical Engineering, Xi’an University of Architecture and Technology, Xi’an 710055, China

**Keywords:** surface acoustic wave resonator, topological insulator, quality factor, insertion loss, sensitivity

## Abstract

This article introduces a novel petal-like SAW topology insulator, which can transmit sound waves with low loss and high flexibility in an ultra-wide frequency band by simultaneously adjusting multiple structural parameters of phononic crystals. Using finite element analysis, it was found that adjusting these parameters can generate a broadband gap of 55.8–65.7 MHz. This structure can also achieve defect immunity and sharp bending in waveguide transmission. When this topology insulator is applied to resonators, compared to traditional designs, the insertion loss is reduced by 22 dB, the on-load quality factor is increased by 227%, the off-load quality factor is increased by 1024.5%, and the quality sensitivity is improved by 3.7 times compared to bare devices.

## 1. Introduction

Since the introduction of surface acoustic waves (SAWs, Surface Acoustic Wave), various types of SAW devices have been widely used in the fields of radar, communication and navigation [1,2]. The performance of SAW resonators (such as quality factor Q, insertion loss and sensitivity) directly determines the indexes of the filters, so it is crucial to design and study the high-performance SAW resonators [3].

Numerous scholars have investigated various methods to improve the performance of SAW resonators [4,5,6,7,8]. In 2018, Kimura introduced the SAW resonator substrate structure as *SiO*_2_*-Pt* instead of *SiO*_2_*-AlN* that better confines the acoustic field energy to the surface of the piezoelectric layer, and the Q of its prepared resonator at a specific frequency was dramatically improved [4]. In 2021, Liu et al. proposed a new SAW resonator substrate structure based on an *ALScN* (aluminum nitride scandium-doped) thin film SAW resonator; by varying the film Euler angle and film thickness, it is able to regulate the acoustic wave modes excited by the piezoelectric layer, and it can be made into a high Q resonator [5]. In 2022, Xu et al. proposed a suspended pseudo-electrode array on piezoelectric film, which concentrates the energy in the center region of the SAW resonator, reduces the lead resistance, and significantly increases the quality factor Q [6]. In 2023, Yao et al. proposed a SAW resonator based on a piezoelectric film with an asymmetrically planar rotation angle by utilizing virtual electrodes, which suppressed the energy leakage caused by the non-zero power flow angle and thus improved the Q value of the resonator [7]. Most of the studies were conducted to improve the performance of SAW resonators by changing the structure of the SAW resonator itself or the material of the substrate.

Phononic crystals (PNCs) as a new type of artificial structures/materials, which enable the precise manipulation of acoustic and elastic wave propagation and dissipation [8,9,10,11]. In 2014, Fleury et al. combined phononic crystals with the topological energy band theory in condensed matter physics, constructed a toroidal flow field to break the time-reversal symmetry of acoustic systems, realized acoustic topological states, and proposed an acoustic topological insulator (topological phononic crystals) [12]. In 2016, He et al. realized the energy band inversion at the Dirac point by changing the ratio of the metal rod radius to the lattice constant and performed an experimental demonstration of acoustic topological insulators, which successfully proved the robust unidirectional edge acoustic transport properties of topological insulators [13]. Yu et al. experimentally demonstrated an artificial phononic graphene, which was customized for surface phonons on a LiNbO_3_-integrated platform. The system exhibits transmission characteristics similar to Dirac quasi-particles, especially the pseudo-diffusion phenomenon at Dirac points, resulting in a time beat frequency effect of transmission pulses similar to the Zitterbeiwegung effect. This study takes an important step toward realizing graphene quantum simulators on chips and unique single-chip electroacoustic integrated circuits [14]. In 2017, Lu et al. successfully demonstrated the unidirectional edge acoustic transport characteristics of acoustic topological insulators by rotating the triangular holes inside the honeycomb lattice at different angles to construct two topological insulators with different properties, realizing acoustic waveguides under sharp bends and verifying the topology-protected property for acoustic waves [15]. In 2019, Wang et al. constructed topology-protected surface acoustic waveguides by arranging arrays of air cylindrical holes of a honeycomb lattice on a rigid substrate, verifying the low-loss transmission of acoustic surface waves by the topological insulators [16]. In 2021, Luo et al. arranged rectangular tabs of a square lattice on an elastic plate and constructed topological insulators topologically protected against elastic waves by varying the different heights of the rectangular tabs within a single cell, verifying the unidirectional waveguide and defect-immunity properties for elastic waves [17]. The excellent properties of unidirectional waveguiding, low-loss transmission and the defect immunity of topological insulators make them widely used in wave modulation and acoustic transmission [13,16,17].

The wave modulation property of the topological insulator is applied to the acoustic surface waves excited by the interdigital transducer (IDT) in the SAW resonator, and the high-transmittance transmission of the resonator is realized by waveguiding the acoustic surface waves [18,19]. In 2021, Zhang et al. introduced topological insulators into the SAW resonator, and the sparseness of the copper pillars of the arrays between the IDTs at the two sides is changed to realize the SAW topological insulators, and the topological interface is constructed on the SAW resonator to realize the overall transmittance improvement [18]. In 2022, Wang et al. changed the radius size of the honeycomb micropillars to construct two SAW topological insulators with different properties so that the transmittance of the SAW resonator is greatly improved [19]. However, the above SAW topological insulators have narrow energy band gaps and small resonator operating bandwidths, resulting in the poor adaptability of SAW resonators [20].

In this paper, a class of petal-shaped SAW topological insulators is proposed for improving the performance of SAW resonators. The acoustic propagation properties of the topological insulator are utilized to achieve the high performance of the SAW resonator with low loss, high robustness, and high flexibility in acoustic wave transmission under ultra-wide bandwidth. In the first part, the effect of multistructural parameters on the energy band structure is investigated, the energy band inversion and the topological phase transition of the system is analyzed, and the topological insulator formed by the change in multistructural parameters is determined by combining with Chern number calculations. In the second part, the properties of topological insulators are investigated, and the interface propagation, defect-immunity propagation, and sharp bending waveguide properties of topological states of topological insulators are verified. In the third part, the topological insulator is applied to an SAW resonator, its S21 parameters are tested by finite element analysis, and its sensitivity and Q are calculated. Finally, a short conclusion is presented.

## 2. SAW Topology Insulator Design and Energy Band Characterization

### 2.1. Unit Cell Model of Phononic Crystals

**Figure 1:**
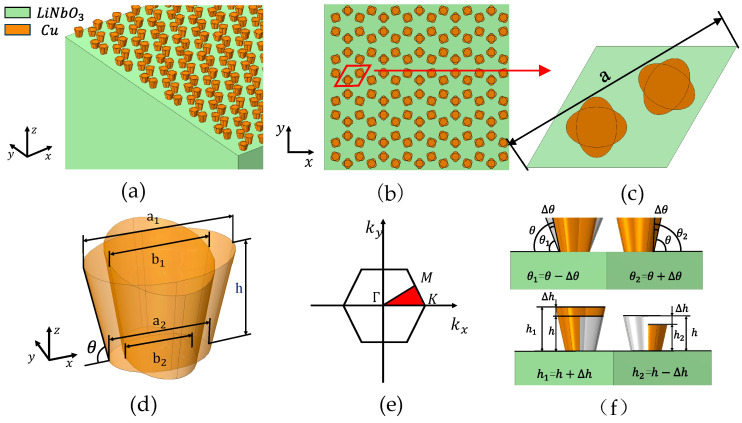
(**a**) Schematic diagram of a phononic crystal, a honeycomb lattice consisting of identical small copper pillars on the *LiNbO*_3_ half-space; (**b**) phononic crystal plate in the plane view; (**c**) top view of petal-shaped lattice; (**d**) structural dimensions of the petal-like copper pillars; (**e**) first Brillouin zone and the irreducible Brillouin zone of the unit cell (red region); (**f**) schematic diagram of the altered geometrical parameters of the copper pillars.

To facilitate electrical pumping and the transduction in SAWs, half-space substrates are usually piezoelectric, such as lithium niobate (*LiNbO*_3_) [21,22]. In this paper, a semi-infinite substrate *LiNbO*_3_ on a periodic honeycomb arrangement of petal-like copper (*Cu*) pillars is shown in Figure 1a, with green representing the *LiNbO*_3_ substrate, and yellow representing the copper pillars, in which the periodic copper pillars on these *LiNbO*_3_ periodic copper pillars on the substrate are called phononic crystals. Figure 1b shows a plane view of the structure, where the diamond-shaped red box represents the original cell of the phononic crystal. The enlarged top view of the original cell is shown in Figure 1c, where the lattice constant a = 45 μm, and each copper pillar is located at the two thirds of the diagonal of the original cell (i.e., the center of each copper pillar is located at the center of gravity of the corresponding triangle in the diamond). The dimensions of the copper pillars are shown in Figure 1d and consist of two identical concentric elliptic cones vertically interlaced to form a petal-like structure, where the upper ellipse long axis a_1_ = 11.25 µm and the lower ellipse long axis a_2_ = 7.5 µm; each ellipse maintains the ratio of short and long axes *u* = a_1_/b_1_ = 1.5, the height of the copper pillar is *h* and the taper angle is *θ*. Figure 1e shows the first Brillouin zone corresponding to this unit cell, and the red region is the irreducible Brillouin zone.

**Figure 2:**
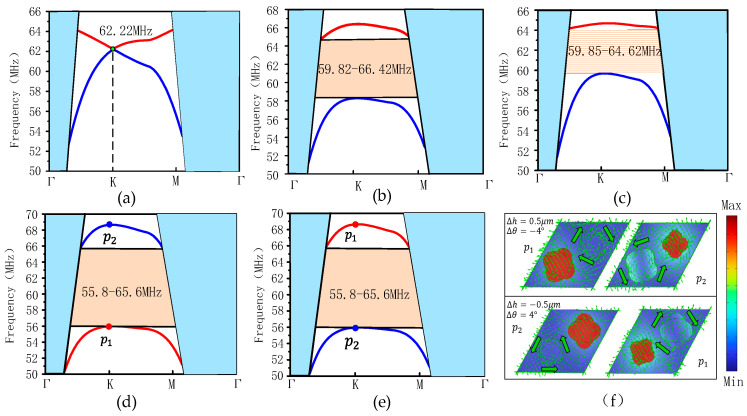
(**a**) Diagram of the energy band structure of the phonon crystal at *θ* = 76.8°, Δ*θ* = 0°; *h* = 8 µm, Δ*h* = 0 µm, with a frequency of 62.22 MHz at the Dirac point (green dot); (**b**) energy band structure diagram at Δ*θ* = 4°; Δ*h* = 0 µm; (**c**) diagram of the energy band structure at Δ*θ* = 0°; Δ*h* = 0.5 µm; (**d**) diagram of the energy band structure at Δ*θ* = 4°; Δ*h* = −0.5 µm; (**e**) diagram of the energy band structure at Δ*θ* = −4°; Δ*h* = 0.5 µm; (**f**) The modes *p*_1_,*p*_2_ in the energy band, the mechanical energy fluxes are green arrows.

When the copper pillar height *h* = 8 µm, taper angle *θ* = 76.8°, we use the finite element software Comsol Multiphysics 6.0 to calculate the structure. The band structure of the phononic crystal is shown in Figure 2a. It can be seen that due to the C_6v_ symmetry of the structure itself, a degenerate Dirac point (green point in the figure) can appear at the corner K of the Brillouin zone with a frequency of 62.22 MHz.

SAW topological insulators can be obtained by breaking the symmetry of the phononic crystal system [23,24]. Therefore, changing the structural parameters of the two copper pillars to break the lattice symmetry and destroy the symmetry of the system can lead to the formation of SAW topological insulators with topological properties. Keeping other parameters constant, changing the taper angle *θ* of the two copper pillars, the upper surface petals are scaled proportionally as shown in Figure 1f, where *θ*_1_ = *θ* − Δ*θ, θ*_2_ = *θ* + Δ*θ*. When Δ*θ* = 4°, the energy band structure is shown in Figure 2b. At this time, the Dirac point at the K point is opened, and the band gap range is 59.82–66.42 MHz.

Further, while keeping other parameters constant, the height *h* of the two copper pillars is varied, and the upper surface petals are then changed as shown in Figure 1f, where the *h*_1_ = *h* + Δ*h, h*_2_ = *h* − Δ*h*. When Δ*h* = 0.5 µm, the energy band structure is shown in Figure 2c; at this time, the Dirac point is also opened and the band gap range is 59.85–64.62 MHz.

Figure 2b,c show that varying a parameter alone (*θ* and *h*), the phononic crystal energy bands exhibit band gaps of different widths. Further, it was investigated that when multiple structural parameters are varied (*θ* and *h* simultaneous changes), there are changes in the energy band structure and band gap. When Δ*θ* = 4°; Δ*h* = −0.5 µm, the energy band structure diagram is shown in Figure 2d. At this time, the Dirac point at the K point opens, and a wide band gap of about 10 MHz appears with a band gap of 55.8–65.7 MHz, which is 50% wider than the band gap for single-parameter variations (at Δ*θ* = 4°) and 107% wider than the band gap for single-parameter variations (at Δ*h* = 0.5 µm).

### 2.2. Energy Band Properties of Phononic Crystals

Due to the generation of Dirac points in the above energy band structure, the opening–closing–opening of Dirac points can be realized by parameter changes, the energy band inversion phenomenon appears, and the topological phase transition of the topological insulator system is realized by using the energy band inversion phenomenon, which is the prerequisite for the realization of the topological boundary states [13]. Therefore, we take the structural parameter values of the original unit cell as the intermediate state (i.e., the height and taper angle at which the Dirac point is generated). When the change in structural parameter values crosses from one side of the intermediate state to the other, the band curve will change, exhibiting the band inversion achieved by the opening and closing of the Dirac point. The phononic crystal in this study is a phononic crystal with C_6V_ symmetry, and the lattice symmetry is broken by changing the geometrical parameters of the phononic crystal so that the crystal has only C_6_ symmetry, thus realizing a simple and merged Dirac point on its energy band [13,25,26,27].

Figure 2d shows that when Δ*θ* = 4°, Δ*h* = −0.5 µm, the Dirac point opens, and the red band curve is located below the blue band curve. In order to further observe the structural characteristics on the band curve, we selected a mode also located at the corner point k of the Brillouin zone, and the modes of the *p*_1_, *p*_2_ points are shown in Figure 2e. The modal resonance of the energy band where the *p*_1_ point is located occurs in the left copper pillar, and the modal resonance of the energy band where the *p*_2_ point is located occurs in the right side of the copper pillar. And when Δ*θ* = −4°, Δ*h* = 0.5 µm, the energy band structure is shown in Figure 2e. The modes of the two points *p*_1_ and *p*_2_ are shown in Figure 2f, and it is found that the modes resonance sides of the *p*_1_ and *p*_2_ points are unchanged, and the energy band of the *p*_1_ point is above the energy band of the *p*_2_ point. Comparing with Figure 2d,e, it is found that the positions of the energy bands where *p*_1_ and *p*_2_ are located are exchanged up and down, and this energy band inversion process indicates the occurrence of a topological phase transition [28]. A further observation of Figure 2f reveals that the mechanical energy fluxes (green arrows) at the two points *p*_1_ and *p*_2_ show clockwise and counterclockwise distributions, respectively, and therefore, the positive and negative cases of the Chern numbers (C±) associated with the two energy bands are heteroscedastic [28,29].

**Figure 3:**
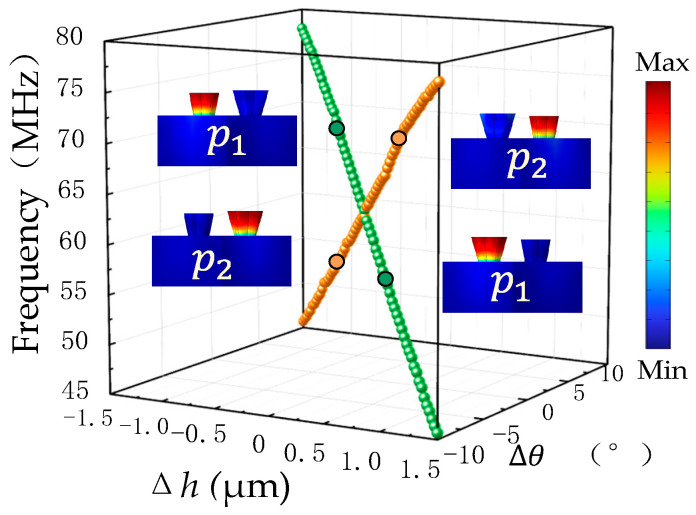
Effect of the variation in the copper pillar parameters on the eigenfrequencies of the energy bands at point K in the Brillouin zone, where *p*_1_, *p*_2_ are modalized at Δθ = 4°, Δh = −0.5 µm versus Δθ = −4°, Δh = 0.5 µm.

In order to clearly show the energy band reversal, the two parameters of copper column angle Δ*θ* and copper column height Δ*h* in the unit cell are scanned in detail. With the simultaneous variation in the lattice parameters Δ*θ* and Δ*h*, the variation process of the energy band positions of the topological multipole modes (*p*_1_, *p*_2_) and the corresponding modes at some points are shown in Figure 3. Observing Figure 3, it is found that the energy band positions of the topological multilevel mode (*p*_1_, *p*_2_) are exchanged up and down once, and there is an energy band inversion in the process of the change in Δ*θ* (−10° to 10°) and the height difference Δ*h* (−1.5 to 1.5 µm).

### 2.3. Interface State Characteristics of Supercell

**Figure 4:**
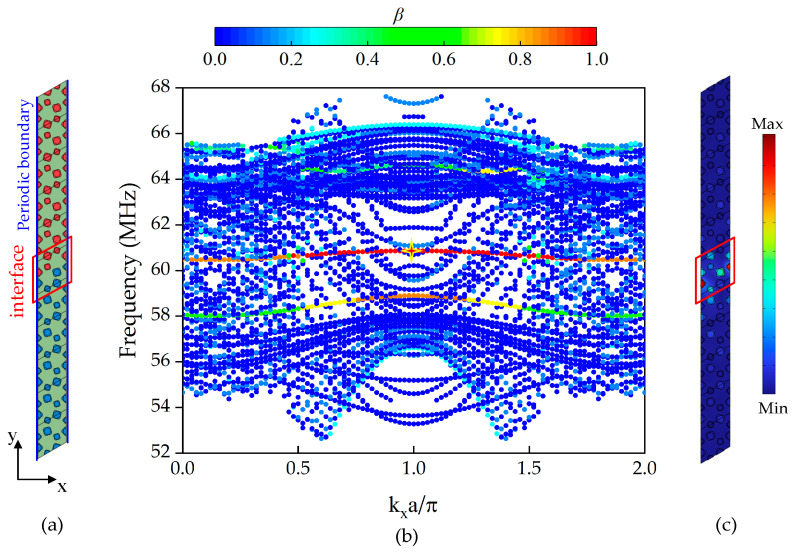
(**a**) Supercell model with changing taper angle; (**b**) energy band curve of supercell; (**c**) displacement mode diagram of special point of structure.

In order to prove that the above structure has a wave propagation mode of interface state in the band gap formed by opening the Dirac point, the supercell structure formed by splicing two different cells with different taper angles (Δ*θ* = ±4°) in Figure 1f is shown in Figure 4a. The interface of the supercell is represented by a red box, and the periodic boundary condition (blue boundary) is set on the long side of the structure, and the rest is set as a free boundary. This is equivalent to scanning a one-dimensional phononic crystal. The energy band curve of the supercell structure is scanned along the *x* direction. As shown in Figure 4b, a parameter *β* is defined to separate the solution of the interface state from all the solutions of the energy band curve, which represents the degree of energy aggregation of the interface state:

(1) $$\beta =\frac{{∭}_{\mathrm{interface}}E\mathrm{dV}}{{∭}_{\mathrm{all}}E\mathrm{dV}}$$

*E* is the kinetic energy density of the structure, which is defined as the integral ratio of the kinetic energy density of the interface state column to that of the whole structure column. When the *β* value is high (red), it is expressed as an interface state mode. A band curve of the interface state can be observed in the band gap of Figure 4b. At the same time, we draw the characteristic mode at *f* = 60.856 MHz, and the interface state can be clearly seen in Figure 4c (the oscillator in the red box in the picture vibrates violently), which provides the possibility that the structure can be robustly transmitted.

## 3. SAW Robust Waveguide Design Based on Topological Boundary States

**Figure 5:**
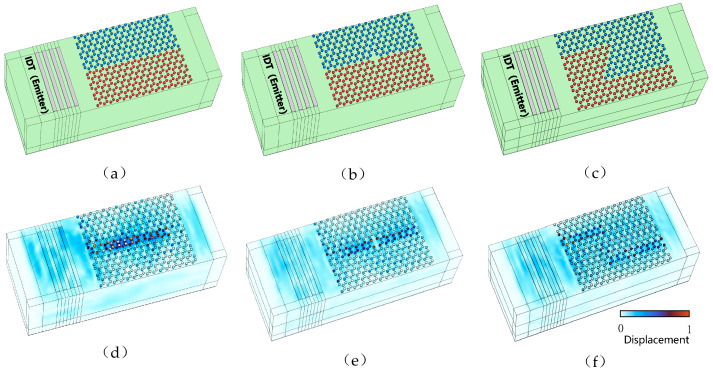
(**a**) Model of “—” channel. (**b**) Model of “—” channel with void defects. (**c**) Model of a “Z” channel with two 120° bends. (**d**) Displacement diagram of the “—” channel. (**e**) Displacement diagram of the “—” channel with void defects. (**f**) Displacement diagram of the “Z” channel.

Since the topological interface states of topological insulators mentioned above allow for the propagation of acoustic waves, for example, along the boundaries of topological insulators, this property essentially suppresses the backscattering of SAWs [16]. SAW waveguides together with the topological interface states counteract a variety of defects, such as the fabrication of transmission paths with defects and as well as sharp bends in SAW waveguide schemes [18]. The construction of topologically protected SAW “—” channels on *LiNbO_3_* substrate (green) using topological insulators is shown in Figure 5a.

Where blue and red colors are used in the model diagram in order to distinguish between different phononic crystals, where blue represents the copper column array with Δ*θ* = −4° and Δ*h* = 0.5 µm, red represents the petal-like copper column array with Δ*θ* = 4° and Δ*h* = −0.5 µm, and the IDT on the left side (in gray) is the input to the topological insulator acoustic wave, which excites the Rayleigh wave with a frequency of *f* = 62.2 MHz. The displacement distribution of the SAW topological insulator is shown in Figure 5d, and it can be seen that the Rayleigh wave excited from the left IDT propagates to the right along the boundary formed by the topological phonon crystal (elastic wave channel) with only little backscattering.

At the same time, if a defect is set up in the “—” channel, as shown in Figure 5b, the topological insulator has a vacancy-deficient defect, and a Rayleigh wave with *f* = 62.2 MHz is excited at the left side of the phononic crystal. The wave can still propagate to the right of the elastic wave channel of the defective “—” channel as shown in Figure 5e, and this strong backscattering suppression and channel defect immunity propagation effect is the result of the non-trivial topology [28]. If a topological insulator is used to form a “Z” channel with two 120° corners as shown in Figure 5c, the robustness of the topological insulator boundaries to sharp curved waveguides is verified. When the IDT excites a Rayleigh wave with the same frequency of *f* = 62.2 MHz, the elastic wave can propagate smoothly along the “Z” channel. The simulated displacement distribution is shown in Figure 5f, which confirms the robustness to sharp bending waveguides. The waveguide nature of this high-quality SAW resonator provides topology-protected applications for future general-purpose SAW filters, multiplexers, modulators, switches, etc. [18].

## 4. SAW Resonator Performance Analysis

### 4.1. SAW Topology Boundary Transmission Effects and S21 Parameters

**Figure 6:**
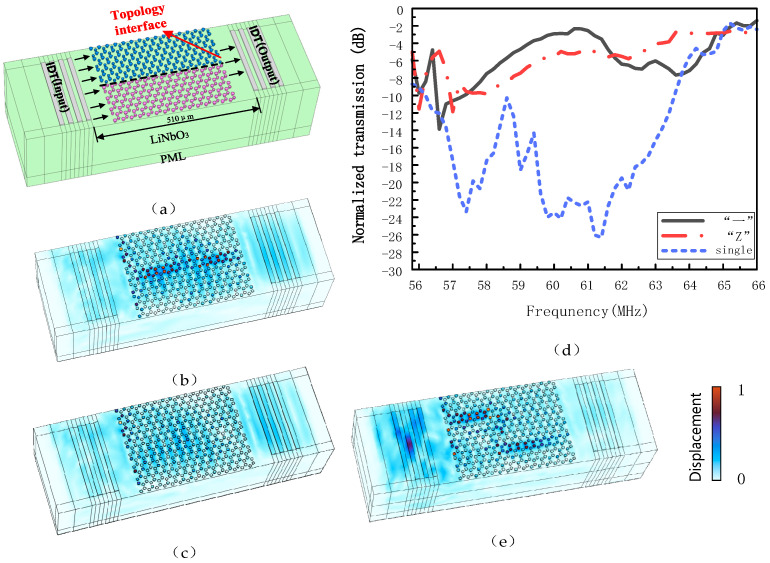
(**a**) Model of “—” channel resonator. (**b**) Simulated transmission displacement plots of the “—” channel. (**c**) Simulated transmission displacement plots for the single copper pillar model. (**d**) Parameter plots of the single copper pillar, the “—” channel and the “Z” channel SAW resonator S21, indicating the transmission effect at different frequencies. (**e**) Simulated transmission displacement plots for the “Z” channel model.

After verifying the interfacial state propagation of the SAW topological insulator, the SAW transmission lines are compared. The above SAW topological insulator is applied to a SAW resonator, in which the resonator substrate is *yz*-90° cut *LiNbO_3_*, the left and right sides of the gray rectangles are the transmitting and receiving IDTs, respectively, and in the middle of the topological insulator is the “—” channel (to distinguish structures with different topological characteristics, we use red and blue colors for differentiation). Figure 6a shows the delay line model of the structure with a logarithmic cross of 2 and a delay distance of 510 μm. At the same time, separate grids are used for the oscillator and substrate to ensure that the maximum grid of each part is less than one fifth of the maximum wavelength. We arranged a perfect matching layer (PML) at the bottom of the model to prevent wave reflection. When the left IDT excited a Rayleigh wave with *f* = 62.2 MHz, the simulated transmission displacement image of the “—” channel of the resonator waveguide is shown in Figure 6b, which clearly shows that the Rayleigh wave propagates in the resonator with strong edge propagation characteristics. For comparison, the rest of the parameters are kept constant, and the simulated transmission displacement when only one type of copper pillar array (Δ*θ* = 4°, Δ*h* = −0.5 µm) is placed between the IDTs on both sides is shown in Figure 6c, where it is clearly seen that with only one type of copper pillar array (single copper pillar), the waveguide scattering is severe. In order to verify the robustness of the topological boundary states to sharp bends, a model consisting of a “Z” shaped topological channel with two 120° sharp bends on the boundary is fabricated, and its displacement diagram is shown in Figure 6e, from which it can be seen that the SAW transmission performance of the “Z” channel model is similar to that of the “—” channel model with strong edge propagation characteristics and little edge scattering.

To further verify the wave propagation properties of the topological insulator, the S21 (insertion loss) of the 55.8–66 MHz surface acoustic wave transmission spectrum was calculated, and the results are shown in Figure 6d. (The calculation formula is T = −20log (P_out_/P_in_), where P_out_/P_in_ is the ratio of front to rear power.) The blue dashed line in the figure indicates the case where there is only a single copper pillar between the two IDTs, and it can be seen that the transmittance decreases significantly after 58.5 MHz. The black solid line indicates the “—” channel between the two IDTs, and it can be seen that the “—” resonator transmits well in the range of 57–66 MHz. Compared to the SAW transmission with a single copper pillar, the insertion loss in the “—” channel is reduced by an average of 22 dB, which fully proves the existence of SAW edge states. The red dotted line indicates the “Z” channel between the two IDTs, and it can be observed that the transmission is also good in the 57–66 MHz range, and the transmittance of the SAW resonator in the “Z” channel is 20 dB higher than that of a single copper pillar resonator on average.

### 4.2. Resonator Quality Factor Q

The lateral leakage and lateral radiation of the SAW mode of operation energy, which in turn affect the *Q* of the SAW resonator, can be reduced by means of a topological insulator [30].

The SAW resonator *Q* is a measure of the energy loss in the resonator and can be defined from the energy point of view as [31].

(2) $$Q=2\pi \frac{q_{1}}{q_{2}}$$

where *q*_1_ is the stored peak energy; and *q*_2_ is the energy consumed per cycle. The most important parameters that reflect the performance of the SAW resonator are the on-load *Q* value denoted as *Q_L_* and the no-load *Q* is denoted as *Q_U_* [32], and the equations are as follows:

(3) $$Q_{L}=\frac{f_{s}}{∆f_{3dB}}$$

(4) $$Q_{U}=\frac{Q_{l}}{1-10^{-\frac{IL}{20}}}$$

where Δ*f_3dB_* is the 3 dB bandwidth, and *IL* is the insertion loss.

**Figure 7:**
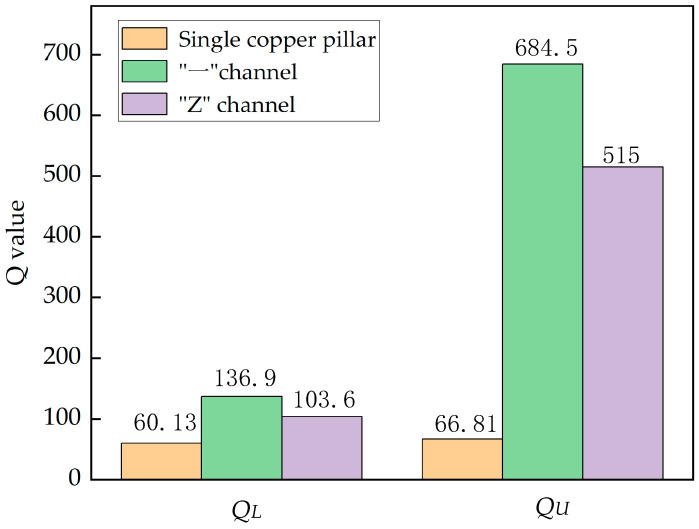
*Q*-value diagrams for “—”, “Z”, and single copper pillar types.

In order to better demonstrate the improvement of *Q* value by applying petal-shaped topological insulators to resonators, we plotted a *Q* diagram based on the above data, as shown in Figure 7, where *Q_L_* is on the left and *Q*_U_ is on the right. When a single copper pillar is placed between the IDTs in the SAW resonator, *Q_L_* = 60.13; *Q_U_* = 66.81. When the “—” channel topology insulator is placed between the IDTs in the SAW resonator, *Q_L_* = 136.9; *Q_U_* = 684.5. Compared to a resonator with a single copper post, the on-load *Q_L_* is improved by 227%, and the no-load *Q_U_* is improved by 1024.5%. When the “Z” topology insulator interface is placed, the *Q_L_* = 103.6; *Q_U_* = 515. The on-load *Q_L_* increased by 171% and the no-load *Q_U_* increased by 779.1%.

### 4.3. SAW Resonator Sensitivity Analysis

The resonator sensitivity based on IDT node displacements and element energies is calculated by the following equations [33,34]:

(5) $$S_{m}^{f}=-c_{m}\cdot f^{2}$$

where *c*_m_ is the mass sensitivity factor, which is defined as

(6) $$c_{m}=\pi^{2}\cdot f_{0}\cdot \left[\left(u_{x}^{2}+u_{\begin{array}{c} y \end{array}}^{2}+u_{z}^{2}\right)/U\right]$$

Here, *f* and *f*_0_ are the operating frequency and resonant frequency of the resonator, *u_x_*, *u_y_* and *u_z_* represent the displacements in different directions on the substrate surface, and *U* is the energy-averaged surface density of the resonator surface. When the SAW resonator is excited at the resonance frequency, the two frequency values of *f* and *f*_0_ are equal. According to Equation (6), it can be concluded that increasing the mass sensitivity can be achieved by increasing the center frequency, improving the displacement of the surface particles, and affecting the energy-averaged surface density *U* by which this parameter can be calculated by the finite element simulation of cell strain data [35]. However, due to the complexity of the value of the energy-averaged surface density and the fact that increasing the center frequency leads to an increase in the complexity of the electronics used for detection, the choice was made to increase the mass sensitivity of the resonator by increasing the displacement of the particles on the surface of the substrate and thus the mass sensitivity of the resonator [35].

**Figure 8:**
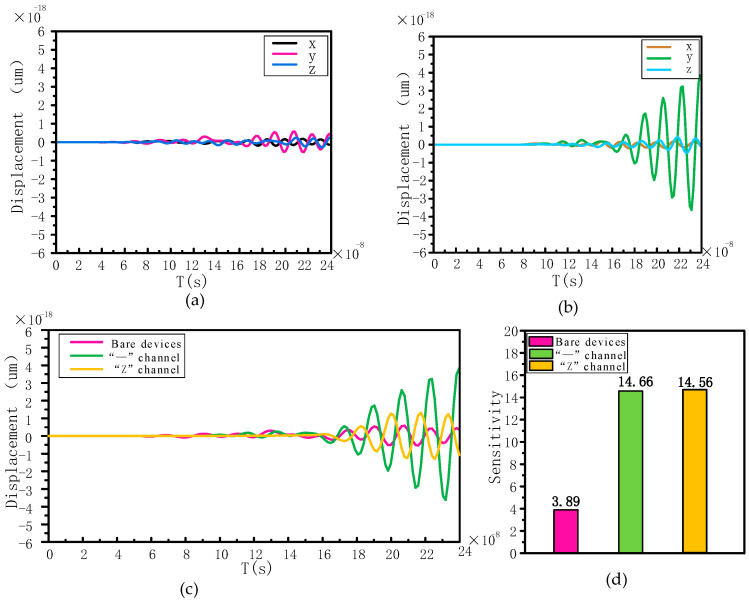
(**a**) Displacement time plots of the bare resonator in direction. (**b**) Displacement time plots of the “—” channel resonator in direction. (**c**) Displacement time plots of the resonators with different fillings of copper pillars (bare, “—” channel and “Z” channel) in the y-direction. (**d**) Mass sensitivity of different filled devices.

Calculate the *x*, *y* and *z* displacements of the nodes on the IDT of the output bare resonator (empty between IDTs on both sides) and the “—” channel topology insulator resonator as a function of time, as shown in Figure 8a,b. Since the resonator substrate is *yz*-90° cut *LiNbO_3_*, for shear horizontally polarized SAW, the primary displacement will occur in the *y*-direction, which is perpendicular to the wave propagation direction with respect to the resonator surface, for a shear horizontally polarized SAW. For the present SAW device, the displacements in the *x* and *z* directions are unimportant relative to the *y* displacement.

In order to compare the displacement changes in the *y*-direction of the resonators with different copper pillar fillings under the same excitation, the displacements in the *y*-direction of the three devices (bare resonator device, “—” channel and “Z” channel) were calculated and plotted versus time, as shown in Figure 8c.

From Figure 8c, it can be observed that when a phononic crystal is placed between the IDTs, the displacement changes in the y-direction are all increased compared to the bare devices. The maximum displacement is obtained for the “—” copper pillar filling with an average amplitude about five times the amplitude of the bare device, and the “Z” filling improves the average amplitude by a factor of 2.5 compared to the bare device.

The corresponding mass sensitivities are calculated based on the displacement amplitudes of the bare, “—” channel and “Z” channel devices, as shown in Figure 8d. Observing Figure 8d, it is found that the bare device has the lowest mass sensitivity of 3.89. The most sensitive device is the “—” channel filled with 14.66, which is 3.7 times more sensitive than the bare device. The “Z” channel filled microcavity device also shows a larger sensitivity of 14.56, which is about 275% higher compared to the bare device.

## 5. Conclusions

In this paper, a petal-like topological insulator applied to SAW resonators is proposed to realize the improvement of resonator performance. By simultaneously changing multiple structural parameters to break the symmetry of the phononic crystal system, the effect of multiple structural parameters on the energy band and the topological phase transition process are analyzed, and the results show that the energy band gap broadens to 55.8–65.7 MHz with the simultaneous change in multiple parameters. Scanning the two parameters, the angle of the copper column (Δ*θ*) and the height of the copper column (Δ*h*), it is found that during the variation in the two parameters Δ*θ,* Δ*h*, the energy band positions of the topological multilevel modes are exchanged up and down once, and there is a one-time energy band inversion. The topological properties are determined by Chern number calculation to form an SAW topological insulator. Based on the waveguide properties of the topological insulator, the obtained SAW topological insulator with an ultra-wide band gap is applied in an SAW resonator to verify the low loss and high flexibility of waveguide transmission. The results shows that regardless of the “—” channel or “Z” channel, the transmission effect is favorable in the range of 59–66 MHz, and the insertion loss is reduced by 22 dB and 20 dB, respectively, compared with the single interface. The quality factor Q has improved greatly with a 227% increase in on-load Q*_L_* and a 1024.5% increase in no-load Q*_U_* for the “—” channel, and a 171% increase in on-load Q*_L_* and a 779.1% increase in no-load Q*_U_* for the “Z” channel. The mass sensitivity is also greatly improved with a 375% increase in sensitivity over the previous bare device in the presence of a petal-like topological insulator. This may pave the way for next-generation high-performance acoustic resonators on a variety of commonly used piezoelectric materials or many emerging 2D piezoelectric materials.

## Data Availability

The original contributions presented in the study are included in the article; further inquiries can be directed to the corresponding author.
